# Multiple Episodes of Severe Bronchospasm During General Anesthesia: A Case Report

**DOI:** 10.7759/cureus.21521

**Published:** 2022-01-23

**Authors:** David Garcia, Mira Kehar, Ejaz S Khan, Roni Mendonca, Michael Girshin

**Affiliations:** 1 Anesthesiology, Metropolitan Hospital Center, New York, USA; 2 Anesthesiology, University of Texas Medical Branch (UTMB) Galveston, League City, USA

**Keywords:** intubation, airway hyperreactivity, general anesthesia, desaturation, allergic reaction, bronchospasm

## Abstract

Bronchospasm is the clinical component of exacerbated underlying airway hyper-reactivity or as part of a more severe underlying pathology such as anaphylaxis. This article reports multiple episodes of bronchospasm after general anesthesia induction for elective surgery of laparoscopic cholecystectomy. Bronchospasm is a joint event during the intubation period, especially in patients with respiratory disease, but in most cases resolves without further complications. It was manifested in isolation in this patient, with no cardiovascular compromise, no skin signs. This case report reviews the literature and the algorithm taken to manage this adverse event and guarantees patient care and successful outcome.

## Introduction

Bronchospasm is a type of airway hyper-reactivity triggered by an underlying respiratory disorder. This case was observed in a patient with respiratory disease who underwent general anesthesia. It can be identified by extended expiration, wheeze, and increased peak airway pressures. If not treated, it may lead to hypoxia, hypotension, and increased morbidity and mortality [1]. An uneventful procedure is critical to a patient's well-being. The management of bronchoconstriction should be continuous and focused on the underlying etiology. Monitoring for clinical signs and symptoms of bronchoconstriction should remain during the procedure and at any phase of the anesthetic period. This case report aims to discuss the critical aspects of intraoperative bronchospasm.

## Case presentation

The patient was a 31-year-old Hispanic female, 60 kg, American Society of Anesthesiologists (ASA) II with a past medical history of ectopic pregnancy, was treated with methotrexate, and was admitted with right upper quadrant pain for laparoscopic cholecystectomy. The patient had no other past medical or surgical history, and all preoperative investigations were within normal limits, except for mild transaminases elevation, no recent upper respiratory tract infections, or smoking. No abnormalities were found on cardiac and pulmonary physical exams. Our patient was taken to the operating room; ASA monitors were placed, and general anesthesia was induced by IV injections of midazolam 2 mg, fentanyl 100 mcg, lidocaine 60 mg, propofol 200 mg, and rocuronium 40 mg. An endotracheal tube (ETT) was placed on the first attempt, and placement was confirmed by auscultation and capnography readings. The patient was ventilated with volume-controlled ventilation, a tidal volume (TV) of 400 mL, a respiration rate of 12, FiO2 0.4, and sevoflurane was used for anesthesia maintenance.

The patient received a slow push of cefazolin 1 g intravenously, and pneumoperitoneum was created. Subsequently, the pulse oximetry started trending down, and the 'shark-fin' capnography pattern was observed. The FiO2 was increased from 0.4 to 1, and manual ventilation was started. However, pulse oximetry rapidly continued to decrease to 60%. At this point, pneumoperitoneum was released, and the anesthesia provider requested help. Two other anesthesia providers present as SpO2 continued to decline to 44%. Severe bilateral wheezes were auscultated, no signs of allergic reactions were noticed, blood pressure and heart rate were within normal limits, suggesting severe bronchospasm. On monitors, minute ventilation (MV) dropped from 4.1 L/min to 0.2 L/min, TV from 400 mL to 18 mL, and mean airway pressure (Pmean) increased from 10 cm H2O to 38 cm H2O. Given the severity of the bronchospasm, the anesthesiologist ahead of the case decided to administer epinephrine 200 mcg IV bolus, and no immediate improvement was observed after 5 min. The initial dose of epinephrine was followed by two other doses of 200 mcg IV, along with eight puffs of albuterol inhaler via ETT because the patient still had the same wheezes pattern on physical exam and increased Pmean with low TV and MV. Dexamethasone 10 mg IV and diphenhydramine 25 mg IV were also administered. These interventions yielded only a transient increase in SpO2. Therefore, it was decided to exchange the ETT due to a possible airway hyper-reactivity caused by ETT, cuff herniation, tube kinking, or mucus plug, and the ventilator could not deliver the set TV with elevated mean airway pressure of 38 cm H2O. Direct laryngoscopy was performed to examine the airway; during this time, mild to moderate edema was noticed on epiglottis, vocal cords, and arytenoids; due to edema, the anesthesiologist decided to reintubated the trachea to secure the airway instead of using a laryngeal mask. Right after ETT placement, there was an immediate improvement of Sp02 to 100%, bilateral airway entry equally, MV 3.8 L/min, TV 398 mL, and Pmean 14 cm H2O. After a discussion with the surgical team, it was decided to continue the surgery. After the first episode of bronchospasm, the patient was kept under deep anesthesia with sevoflurane 4%, and no other drugs were administered. Twenty minutes past the first episode of desaturation and tube exchange, when the gallbladder was ready to be incised, the patient began to desaturate with new onset of wheezes and dynamic changes on TV 98 mL, MV 1.1 L/min, and Pmean 32 cm H2O, SpO2 dropped to 66%. The inspiration fraction of oxygen was increased to 100%, albuterol eight puffs were administered via ETT, and epinephrine 100 mcg IV, two doses were given. After the second dose of epinephrine, SpO2 99%, TV 380 mL, MV 4 L/min, Pmean 14 cm H2O, the patient recovered. The anesthesiologist discussed the option to abort the procedure due to the repetition of severe bronchospasm and patient safety; however, since the gallbladder was mobilized for incision, the surgical team requested 15 min to finish the procedure, and the anesthesiologist agreed to continue with the surgery.

As the surgical team was suturing skin, approximately 10 min after the second bronchospasm episode, the patient desaturated for the third time. The patient received one dose of epinephrine 100 mcg intravenously and recovered saturation from 79% to 100%, TV from 180 mL to 398 mL, MV from 2.1 L/min to 4.4 L/min, and Pmean from 29 cm H2O to 11 cm H2O. At this stage, a plan was formed to perform deep anesthesia extubation due to the possibility of airway manipulation hyper-reactivity caused by ETT. The extubation was managed, and a laryngeal mask airway (LMA) was placed to maintain the patient under ventilation until fully awake. A blood sample was obtained and sent to a laboratory for tryptase studies to rule out an allergic reaction. The patient emerged from anesthesia with stable vital signs, and the LMA was removed, no signs of respiratory distress were noted, bilateral air entry without wheezes. The patient was hemodynamically stable and transferred to the intensive care unit (ICU).

While in the ICU, a chest X-ray was ordered and showed mildly diffusely prominent vascular and interstitial markings, mild peribronchial cuffing, and no focal infiltrates; another episode of bronchospasm was reported and treated with albuterol and ipratropium nebulizer. The pulmonary team evaluated and assessed the patient, a diagnosis of asthma/reactive airway was made and recommended budesonide and formoterol inhaler and pulmonary function tests. On postoperative day 1, a second blood sample for tryptase study was obtained. Both blood samples for tryptase studies were negative, 2.4 ug/L and 2.2 ug/L, respectively, confirming intra-operative bronchospasm episodes. Forty-eight hours later, the patient was discharged from the hospital with pulmonary, allergy, and immunology follow-ups. The patient has followed up primary care physician, currently being treated with budesonide and formoterol inhaler, but refused pulmonary follow-up, allergy, and immunology studies.

## Discussion

Bronchospasm is a condition characterized by increased airway hyper-reactivity. It can cause severe respiratory distress and frequent coughing and wheezing. The sudden onset of bronchospasm after anesthetic induction is linked to various physiological changes, with cardiovascular changes and skin signs clinically suggesting a drug-induced anaphylactic response [1]. Bronchospasm commonly occurs during the perioperative period following an anesthesia induction or intubation. They can be triggered by various factors, such as an immediate hypersensitivity reaction including IgE-mediated anaphylaxis, a non-pharmacological mechanism, or pharmacologic-induced by histamine-releasing drugs in patients with uncontrolled airway hyper-reactivity [1-7].

The complex mechanism that causes bronchoconstriction is caused by various factors, such as the airway nerves responsiveness, smooth muscle, epithelial tissue, and inflammatory cells surrounding the respiratory tract triggering an afferent signal to the brainstem, and from there an efferent signal through the vagus nerve leading to acetylcholine release in the airway. Moreover, that is why antimuscarinic drugs can help prevent or treat this condition because they will counteract the acetylcholine on M3-muscarinic receptors that cause bronchoconstriction. Also, noncholinergic neurotransducers are capable of releasing tachykinins and vasoactive intestinal polypeptides. They can also stimulate the release of procontractile neuropeptides.

Studies show that propofol preferentially reduces tachykinin-caused airway constriction in patients with respiratory distress. It has been known that deep anesthesia can modulate and attenuate tachykinin-caused bronchoconstriction in patients with respiratory distress. Despite these protective effects of IV propofol and the adequate induction dose used in the current case, reflex-induced bronchoconstriction may still be triggered in patients who had previously unrecognized and untreated asthma.

Before surgery, a comprehensive evaluation of pulmonary risk factors should be performed to identify the potential complications of an asymptomatic or poorly controlled airway hyper-reactivity. It is well known that perioperative and postoperative complications depend on the severity of asthma at the time of surgery, type of surgery, and anesthesia techniques. 

If patients with asthma have a history of uncontrolled use of inhaled corticosteroids and/or 2-agonists, recent use of systemic corticosteroids, recent exacerbations, emergency department, recent hospital visits, and intubations, they should be assessed for poor control. Same as wheezing, cough, increased sputum production, shortness of breath, and diurnal variability in peak expiratory flow rate indicate further assessment [7]. These guidelines mainly focus on the management of asthma before surgery. It is considered a leading cause of bronchoconstriction during surgery and should be suspended until the patient is optimized, and their medication should be continued until the time of surgery.

Ideally, patients should be counseled to stop smoking before surgery. It is also possible to postpone surgery if the patient has an upper respiratory tract infection and the complete resolution of symptoms is not achieved, especially children who have an increased risk of bronchospasm, so it may be necessary to postpone surgery for two to three weeks after the complete resolution of symptoms. Pretreatment with β2-agonist, 30 min prior to surgery, propofol for induction of anesthesia, and adequate depth of anesthesia before airway manipulation reduce the risk of bronchospasm [7]. Regional techniques and general anesthesia with LMA can help minimize the risk of bronchospasm and avoid airway manipulation if indicated.

Understanding the physiological mechanism of a hypersensitivity reaction's clinical signs and symptoms timeline is crucial. Perioperative anaphylaxis caused by neuromuscular blockers has initially cardiovascular signs that befall within minutes after the drug injection and may be associated with or followed by bronchospasm [1]. Cardiovascular disturbance is the hallmark of severe IgE-mediated anaphylaxis. Latex-induced anaphylaxis usually occurs in patients with prior latex exposure, a cross-reaction food allergy, or atopy. The event usually takes about 60 min after surgery to develop because latex is slowly absorbed. The cause of the hyper-responsiveness may be different from the usual causes, such as an ETT or the suction catheter, without antigen exposure. It is essential to keep in mind that positive end-expiratory pressure with bronchospasm can reduce venous return and cardiac output, plus the association of hypoxia and respiratory failure from inadequate ventilation may lead to cardiovascular collapse [1].

Differential diagnosis

Bronchospasm usually occurs during anesthesia's induction and maintenance phases and is less frequently faced in the emergence and recovery phases. It can be triggered by a severe allergic reaction or anaphylactic reaction during the maintenance stage. The most common causes of bronchospasm are usually related to endotracheal intubation. When evaluating bronchospasm, we should consider relevant differential diagnoses and contributing factors such as mechanical obstruction, laryngospasm, inadequate depth of anesthesia, and drug induced.

In unexplained bronchospasm, airway soiling due to secretions, regurgitation, or aspiration should be considered. For that, using an LMA or an incompletely inflated cuff or uncuffed ETT should be considered to prevent or minimize the accumulation of secretions or continuous stimulus of the airway [7].

Morbidity and mortality rates

Recent data show that respiratory events estimated for 28% of cases involving anesthesia-related brain damage and death in the United States. In these cases, bronchospasm was included in the other categories and corresponded to 11% of total respiratory issues [1].

Role of tryptase

High tryptase levels can help distinguish anaphylaxis from other conditions for a possible differential diagnosis, like vasovagal reflexes, septic or cardiogenic shock, seizures, benign flushing, or carcinoid syndrome. Tryptase levels increase as fast as five minutes following the clinical onset of anaphylaxis, achieving maximal levels within 30-90 min, and then wane with a half-life of approximately 2 h. 

The tryptase surge is a strong indicator of the severity of hypotension during an anaphylactic reaction, but it can also be seen induced by medications. However, normal levels do not reject anaphylaxis, especially in food-induced reactions, samples obtained more than four hours after initial symptoms and those without hypotension.

A blood sample should be obtained within 15 min to 3 h after an event onset if a person with suspected anaphylaxis is being considered for tryptase studies. The specificity and sensitivity of tryptase studies have not been accurately determined but increase with clinical severity. Significant elevations in tryptase may last for many hours [3].

Treatment

Reverting the bronchospasm and avoiding hypoxemia are the goals of initial treatment. If an isolated bronchospasm occurs, increasing the oxygen concentration to 100% should be initiated, and manual bag ventilation promptly started to assess pulmonary compliance and identify high-circuit pressure causes. Deepening anesthesia may be required because an inadequate depth of anesthesia may lead to bronchospasm induced by ETT placement or airway manipulation. In addition, increasing the inhalational concentration of an anesthetic is also helpful to avoid or reduce respiratory hyper-reactivity, except desflurane because of its airway irritant side effect, especially in asthmatics and smokers.

Mechanical ventilation in acute bronchospasm aims to avoid and correct hypoxemia. Consider reducing tidal to avoid high peak airway pressures and barotrauma. Hypercapnia is tolerated if oxygenation is adequate and if severe acidosis does not occur. Ventilation should include a long expiratory time to allow total exhalation and reduce breath stacking and intrinsic positive end-expiratory pressure (PEEP) because they may increase intrathoracic pressure, decrease venous return, and lead to hypotension [7].

Inhaled β2-agonists are indicated for the quick relief of bronchoconstriction due to the activation of the 2-adrenergic receptors on the airway smooth muscle. They can be given through a nebulizer or metered-dose inhaler.

Ipratropium has been shown to reduce reflex-induced bronchoconstriction with potency similar to inhaled β2-agonists. It can be combined with a β2-agonist drug to treat life-threatening bronchospasm.

Systemic steroids should be utilized to treat respiratory conditions like asthma, chronic obstructive pulmonary disease (COPD), or even bronchospasm because they decrease airway inflammation. They can help decrease the severity of exacerbations by preventing them from happening, and they take up to 6 h before exerting their beneficial effect.

Epinephrine should be considered for patients with severe asthma that has been triggered by IgE-mediated anaphylaxis and is prone to cardiac collapse.

Magnesium sulfate is used to treat severe bronchial asthma by relaxing the bronchial smooth muscles. It can be associated with other drugs or when initial treatment fails.

Ketamine infusions have been reported to treat patients with status asthmaticus successfully. Although the effects of ketamine on the respiratory system are not yet apparent, they seem to cause bronchodilation; it seems like the catecholamine released by ketamine causes bronchodilation. Beneficial results are reported within 30 min to several hours.

Antihistamines - they compete with histamine for the histamine-receptors in the respiratory tract. This causes effects such as the reduction in smooth muscle contraction.

Volatile agents - the activity at inhibitory -aminobutyric acid-A chloride channels or modulating calcium sensitivity of the contractile proteins is why deepening anesthesia with volatile agents prevents or relieves reflex-induced bronchoconstriction, except desflurane due to its airway irritant side effect, especially in asthmatics and smokers.

Helium-oxygen - the use of helium-oxygen mixed with oxygen is commonly used for severe asthma exacerbations that are unresponsive to standard therapy or appear to have a component of upper airway obstruction. However, this method alone cannot be recommended due to conflicting data about efficacy. 

Extracorporeal life support oxygenation -this can be helpful in patients with severe asthma/bronchospasm who are with severe asthma refractory to routine care, respiratory acidosis, and mechanical ventilation. However, evidence-based clinical trials are lacking.

Postoperative care and follow-up

A chest radiograph should be ordered if the symptoms are severe and persistent. A chest evaluation should also be performed to rule out pulmonary edema and pneumothorax. If necessary, pharmacological and chest physiotherapy should also be provided. If the condition worsens or the patient has anaphylactic shock, follow-up examinations should be performed [7].

The anesthesiologist must make sure that the patient is referred to an immunological and allergy center for further investigation. The patient, surgeon, and primary care physician should also be informed.

## Conclusions

For a patient under general anesthesia, bronchospasm can become a life-threatening situation. Although other parts that lead to airway inflammation in asthma are many and still not fully understood, factors triggering perioperative bronchospasm are well known. The science efforts could enhance the prevention and treatment of bronchospasm associated with anesthesia. Understanding the physiological mechanism of a hypersensitivity reaction, the clinical signs and symptoms timeline is crucial. In this presented case, the severity of symptoms and dynamic changes on the ventilator were paramount in guiding the intraoperative treatment. It is important to have in mind that following an episode of bronchospasm, other episodes may happen intraoperatively, and the anesthesiologist must be prepared to make decisions focused on patient’s safety. That is why we believe that any anesthesia provider must understand what bronchospasm is, its pathophysiology, differential diagnosis, and algorithm of treatments to provide the best patient care and successful outcome.
